# Supplementary figures and images for: A novel method of screening combinations of angiostatics identifies bevacizumab and temsirolimus as synergistic inhibitors of glioma-induced angiogenesis
Source: PLoS One. 2021 Jun 2;16(6):e0252233. doi: 10.1371/journal.pone.0252233 (PMC8172048; doi:10.1371/journal.pone.0252233)

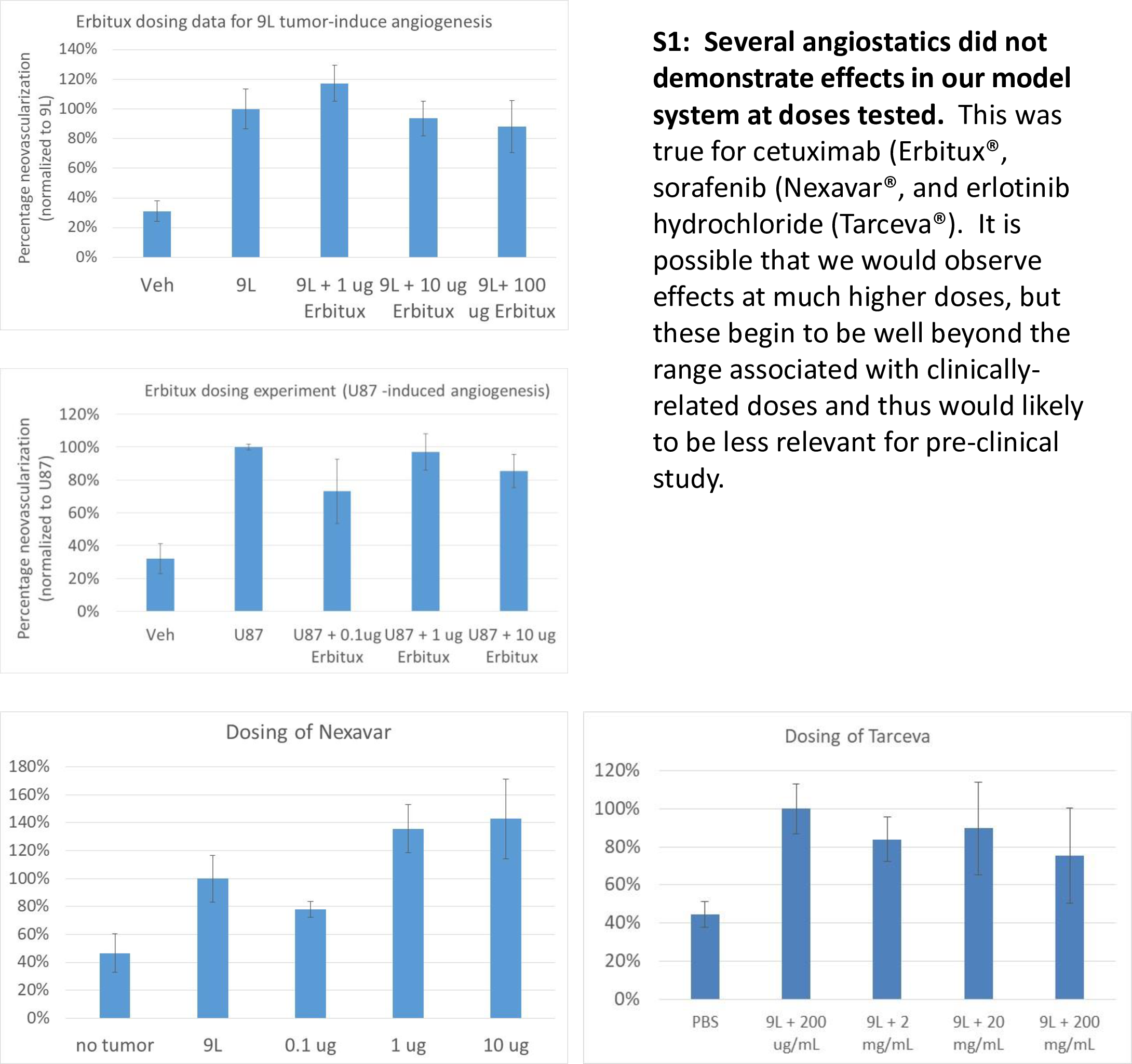

Supplement: S1 Fig — This was true for cetuximab (Erbitux®), sorafenib (Nexavar®), and erlotinib hydrochloride (Tarceva®). It is possible that we would observe effects at much higher doses, but these begin to be well beyond the range associated with clinically-related doses and thus would likely to be less relevant for pre-clinical study. (TIF) [file pone.0252233.s001.tif]

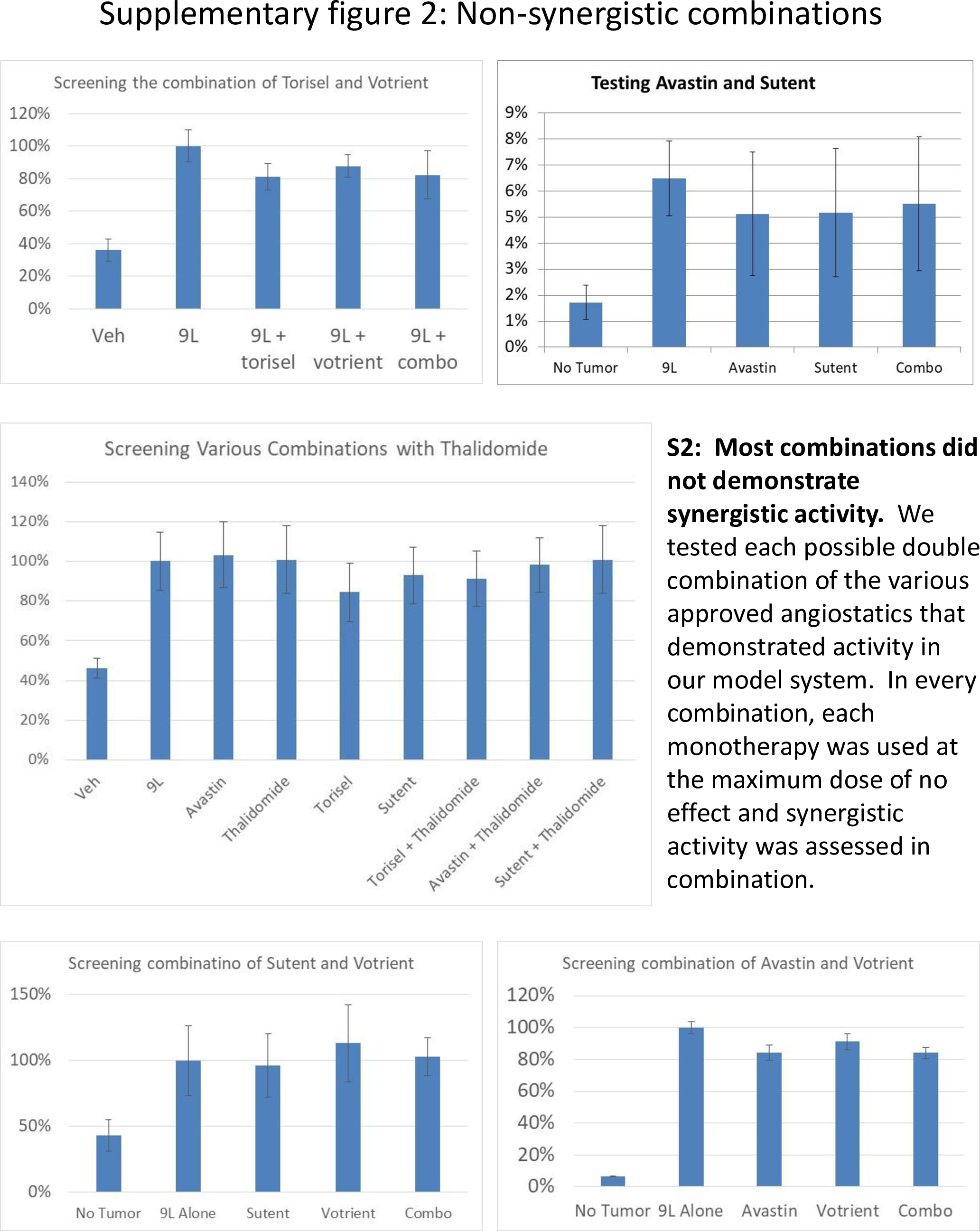

Supplement: S2 Fig — We tested each possible double combination of the various approved angiostatics that demonstrated activity in our model system. In every combination, each monotherapy was used at the maximum dose of no effect and synergistic activity was assessed in combination. (TIF) [file pone.0252233.s002.tif]
