# Supplementary material for: A novel method of screening combinations of angiostatics identifies bevacizumab and temsirolimus as synergistic inhibitors of glioma-induced angiogenesis
Source: PLoS One. 2021 Jun 2;16(6):e0252233. doi: 10.1371/journal.pone.0252233 (PMC8172048; doi:10.1371/journal.pone.0252233)
Supplement: Attachment — Submitted filename: 5.5.21_Response to reviewers R2.docx [file pone.0252233.s004.docx]

Reviewer #1: A minor comment:

A repeated high dose of bevacizumab administration could induce antibodies against to bevacizumab in Rat. So it may not be efficient at later time points in the study. the authors could discuss this issue as well.

While this is a good thought, we are not using a rat model to test bevacizumab and only included the efficacy in rat model references to demonstrate that the antibody-based drug was effective against rat glioma VEGF, such as that which would be produced by the 9L tumor cells.

Reviewer #2: The readability of manuscript has improved tremendously than previous version. However, there are still some issues describing their results/ sub-headings which makes hard to interpret results clearly. It leads to read whole paragraph/section, and then self-assume its context to interpret results. Here are some suggestions.
1. Author should change sub-title of results with specific words in context to their result description. It is hard to interpret with generalized/vague statement for examples

• Maintained activity in combination at lower doses (It is not clear for ‘what activity’, ‘which drugs’)
Line 467 has been modified to “Bevacizumab and temsirolimus maintain synergistic activity when doses are reduced 10-fold”

• Dosing of individual monotherapies (what kind/types of monotherapies)
Line 372 has been modified to “Identifying the maximum dose of no effect for individual
angiostatic monotherapies”

• Monotherapy dosing in the CAM model (add name of monotherapy if it is reference to single therapy, is CAM model refer to Tumor angiogenesis here?)
Line 387 has been modified to “Monotherapy dosing of angiostatics in the CAM model”

• Synergistic activity is observed even at 10-fold lower doses (Please clarify in context ‘Synergistic activity’ which types or class of inhibitors)
Line 485 has been modified to “Synergistic activity of bevacizumab and temsirolimus is observed at 10-fold lower doses.”

• Therapy preparations (what types of therapy? Mono or in combinations)
Line 174 has been modified to “Preparation of angiostatics for screening”

• Quantification of Synergy (please define clearly’ Synergy’ which kinds of inhibitor refer here)
Line 274 has been modified to “Compusyn analysis of synergy”

In addition, we altered a few other subheadings to be more specific and represent the main points of each subsection further in line with the reviewer’s suggestions. These are mainly altered in the methods and results sections and can be viewed through the tracked changes.

We also went through the manuscript and attempted to specify “activity” anywhere that was used by adding appropriate reference to “angiostatic activity” or “activity blocking tumor-induced NV.” These minor changes are evident throughout the manuscript.

2. According to their recent response from Authors, ‘For example, our novel modification of the CAM model system allows for automated quantification of tumor vasculature within the tumor onplants rather than the underlying CAM vessels. To our knowledge, this is unique in the various models for assessing and quantifying angiogenesis. Author should clarify about the title reference whether it used in context to normal Angiogenesis or tumor angiogenesis,. It is not clear in the title ‘Automated quantification of angiogenesis’.

Line 341-342 has been modified to “Development and analysis of automated methods to
quantify neovascularization within the tumor onplants”
